# The Status of Intelligent Control Technology for the Working Height of a Crop Harvesting Header

**DOI:** 10.3390/s25206367

**Published:** 2025-10-15

**Authors:** Chenxu Zhao, Feng Wu, Fengwei Gu, Xinsheng Zhou, Yanqin Zhang, Peng Chen, Jiayong Pei, Hongguang Yang

**Affiliations:** 1 Nanjing Institute of Agricultural Mechanization, Ministry of Agriculture and Rural Affairs, Nanjing 210014, China; y00450240249@njit.edu.cn (C.Z.); wufeng@caas.cn (F.W.); gufengwei@caas.cn (F.G.); 17634376740@163.com (P.C.); peijiayong0615@163.com (J.P.); 2 College of Mechanical Engineering, Nanjing Institute of Technology, Nanjing 211167, China; zyq1028@163.com; 3 Suiping Rural Social Development Service Center, Zhumadian 463100, China

**Keywords:** crops, harvesting header, working height sensor, algorithm

## Abstract

As is well known, intelligence and efficiency are important development directions for modern agriculture. The harvesting header, as key components of crop harvesters, have significant implications for achieving intelligent control of their working height, which has a notable impact on reducing harvest loss. To understand the current state of intelligent control technology for the working height of a crop harvesting header, and to explore their application potential, this article provides a relatively systematic literature review. Firstly, we analyzed the structure and principle of the harvesting header of typical grain and oil crops such as rice and peanuts. Secondly, we briefly described the current methods for controlling the working height of the harvesting header. They mainly use two methods: mechanical profiling and electro-hydraulic profiling. Thirdly, we focused on researching and analyzing the measurement methods and control algorithms for the working height of the harvesting header. Finally, we pointed out the problems in the current height control of the harvesting header. These problems mainly include insufficient measurement accuracy of working height in complex terrain, slow response and large delay of working height hydraulic control system, incompatibility between working height control models and strategies, and relatively single working height measurement methods.

## 1. Introduction

Harvesters play an important role in agricultural production [1]. With the advancement of agricultural technology, harvesters are increasingly moving toward larger scale and intelligent designs, playing an increasingly vital role in modern agriculture [2,3]. To meet the growing demand for harvesting, achieve higher harvesting quality standards, and reduce labor intensity, the number of harvesters in use and the investment in them have been steadily increasing year by year [4,5]. However, the automation and intelligence level of harvesters in China remains relatively low, with cumbersome operations that heavily rely on the artificial experience. In the event of a malfunction, this can result in significant delays, affecting the timely harvesting of crops and the subsequent planting of the next crop [6,7].

Harvesting header height adjustment is one of the key elements in the operation of harvesters. Due to the insufficient accuracy and reliability of harvesting header height detection, issues such as missed cutting and missed picking are widespread, resulting in losses that reduce the actual yield of crops and harm farmers’ economic benefits [8,9,10]. Therefore, it is necessary to improve the harvesting accuracy of harvesters to minimize losses [11,12,13].

At present, there has been some research on intelligent control technology for harvesting header both domestically and internationally. But there is a lack of systematic sorting and analysis of the principles and uses of these intelligent control technologies. Moreover, the majority of studies have employed either contact or non-contact height measurement methods in isolation, failing to fully leverage their complementary potential in terms of accuracy, reliability, and environmental adaptability. Under complex operating conditions, such as excessive slopes or abrupt changes in stubble height, a single measurement approach is prone to failure, leading to control system overshoot. This has become a critical bottleneck hindering the implementation of high-precision, robust header height control systems.

To ensure the systematic and reproducible nature of this review, clear inclusion and exclusion criteria were established. Inclusion criteria: (1) Content involving harvesting header height detection (contact, non-contact) or height control algorithms (PID, fuzzy, adaptive, deep learning, etc.); (2) Peer-reviewed journal articles, conference proceedings, patents, or master’s/doctoral theses. Exclusion criteria: conference abstracts, technical briefs, and height detection research outside the agricultural field. Search time span: 2013–2025 (with over 85% of literature from the past five years); databases: CNKI, Web of Science, Scopus, and EI Compendex.

Based on this, we focus on researching contact and non-contact sensing detection methods as well as height control algorithms in the height control of the harvesting header. Systematically analyze their respective progress and performance differences in the adjustment of the working height, laying the foundation for the development of intelligent control systems for high-precision and high-efficiency harvesting header working height in the future.

## 2. Structure and Principle of the Harvesting Header

**Figure 1:**
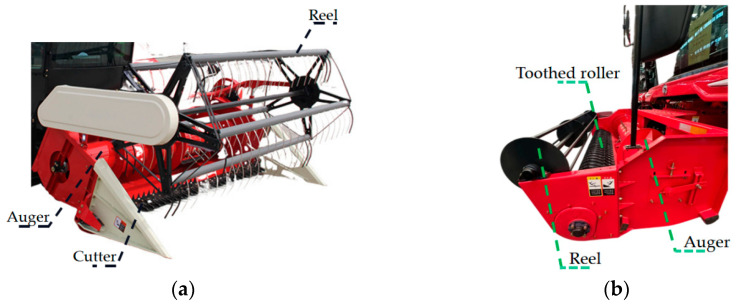
Schematic diagram of the harvesting header. (**a**) Rice cutting header; (**b**) Peanut picking header.

The harvesting header is mainly used in combine harvesters. They can be divided into a cutting header and a picking header. The cutting header is the primary working component of a harvester for harvesting crops such as wheat, rice, and corn. Based on its structure, it can be classified into vertical cutting header, and horizontal cutting header, among others [14,15,16,17]. As shown in Figure 1a, it primarily consists of a cutter, a reel, and a auger [18,19,20,21]. During operation, the reel rotates to guide crops from the field into the cutting area while supporting the stems to maintain an upright position. The cutter severs the crop stalks at high speed. Subsequently, the auger (screw conveyor) transports the cut crops to the threshing mechanism [22,23,24,25].

The picking header is a critical component of crop pickers, used for harvesting peanuts, rapeseed, and similar crops. It is designed to collect crops that have been previously cut or dug and laid out for drying, and to transport them to subsequent processing stages [26,27,28,29]. Based on their structure, they can be classified into spring-tooth drum-type picking header, tooth belt-type picking header, and others [30,31,32,33,34]. As shown in Figure 1b, it primarily consists of a toothed roller, reel, and auger. During operation, the powered tooth roller or belt rotates, enabling its teeth to penetrate and lift the crop mat. The collected crops are then transported via the auger to the shelling or threshing device [35,36,37].

## 3. Classification of Intelligent Control Technology for the Working Height of a Crop Harvesting Header

### 3.1. Mechanical Profiling

Mechanical profiling is commonly used for soybean harvesting [38]. During operation, changes in the height of the harvesting header cause variations in the deformation of the spring plate and the ground pressure exerted by the drag plate [39]. During normal operation, the spring transmits the hydraulic cylinder’s thrust to the harvesting header, with the thrust equal to the header’s self-weight. When encountering convex terrain, the spring tension decreases, causing a drop in hydraulic cylinder pressure. This pressure drop allows the piston to move leftward, which lifts the harvesting header. Subsequently, oil is supplied to the right side of the cylinder, restoring the thrust to balance the header’s weight and returning the spring to its normal state. When encountering a concave surface, the drain valve releases oil, the cylinder piston moves to the right, the spring tension increases, and the cutting header descends until the profiling plate touches the ground. At this point, oil is supplied to the right side of the cylinder again, the spring returns to its normal length, and the thrust equals the weight of the harvesting header [40,41,42].

Researchers both domestically and internationally have conducted studies on mechanical profiling. Shearer [43] installed spring plates at the fixed beam of the header to achieve a flexible connection between the cutter bar and the main body, which permitted the cutting components to undergo vertical elastic displacement. Xing [44] et al. designed a flexible soybean harvesting header that effectively reduces missed cuts, thereby lowering loss rates. Wang [45] et al. proposed a flexible soybean cutting device that uses a spring plate mechanism for flexible connection and angle adjustment, enabling the cutting blade to adapt to changes in ridge height through adaptive deflection, thereby achieving ground-hugging floating cutting. Nie [46] et al. designed a synchronous low-position cutting profiling-following device for soybean-corn strip intercropping. This profiling device follows the profiling plate along the ground’s undulations to ensure precise cutting of the plant’s lower end.

### 3.2. Electro-Hydraulic Profiling

Overall, research on mechanical profiling control has reached a bottleneck due to its limited adaptability and slow response speed, making it suitable only for low-demand operating conditions. In recent years, electro-hydraulic profiling has developed rapidly, offering greater potential and application space.

Electro-hydraulic profiling is the core technology used in modern harvesters to achieve automatic terrain following the harvesting header [47,48]. The profiling systems primarily consist of height measurement mechanisms, control units, and actuators. During operation, profiling components and sensors in the height measurement mechanisms collect harvesting header height information and convert it into electrical signals transmitted to the control unit. The control unit processes these signals, integrates them with control algorithms, and subsequently drives the actuators to adjust the header height [49,50,51,52,53].

## 4. Measurement Methods and Control Algorithms of the Working Height of a Crop Harvesting Header

### 4.1. Measurement Methods of the Working Height of a Crop Harvesting Header

**Figure 2:**
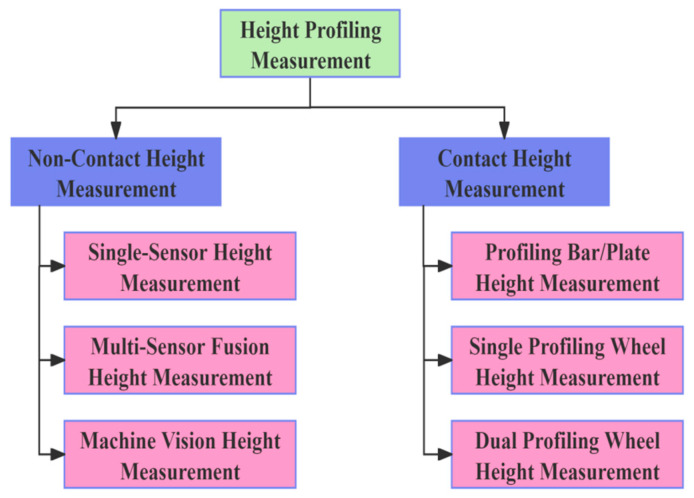
Classification of height measurement methods.

The accuracy of header height measurement, as the input to the entire adjustment system, is critical to its performance The more accurate the detection, the better the subsequent adjustment effect [54]. As shown in Figure 2, domestic and foreign scholars have mainly focused on two aspects of height profiling measurement: non-contact height measurement and contact cutting header height measurement.

#### 4.1.1. Non-Contact Measurement

1. Structure and principle

**Figure 3:**
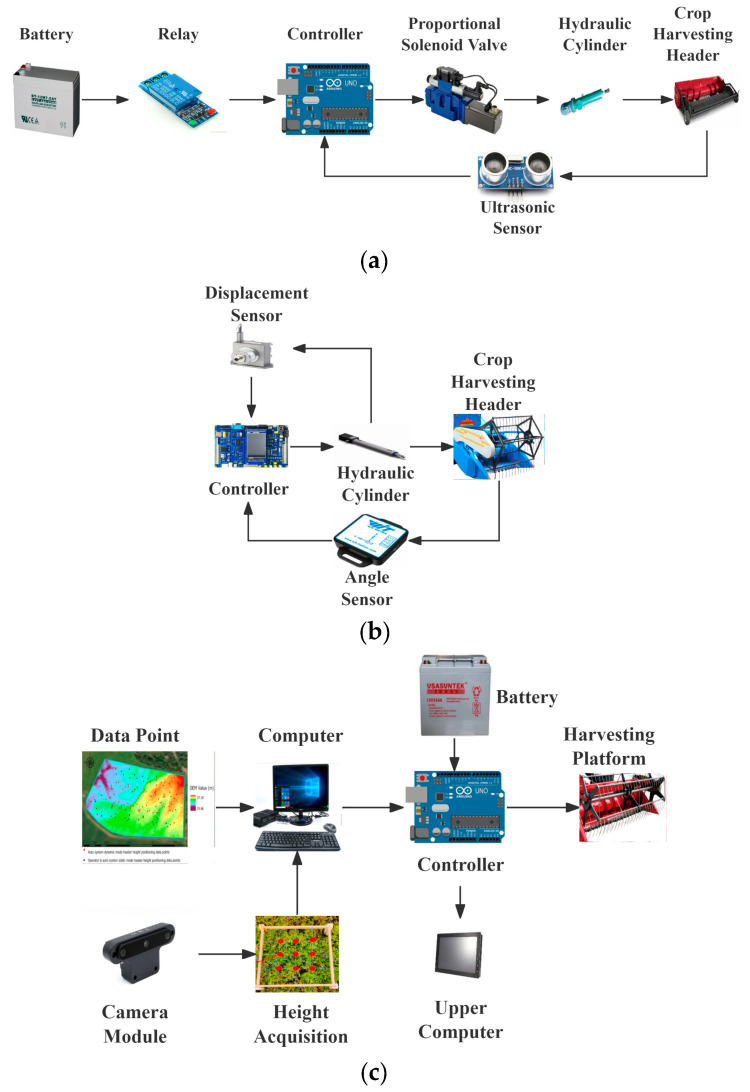
Non-contact height measurement workflow diagram. (**a**) Single-sensor workflow; (**b**) Multi-sensor workflow; (**c**) Machine vision workflow.

Non-contact harvesting header height measurement primarily utilizes sensors or industrial cameras as the measuring mechanism. Based on different hardware configurations, it can be categorized into three solutions: single-sensor, multi-sensor fusion, and machine vision. Figure 3a and Figure 3b illustrate the workflow diagrams for single-sensor and multi-sensor fusion systems, respectively. Sensors capture data from the harvesting header or actuators (hydraulic cylinders) and feed it back to the controller. The controller then outputs appropriate control signals based on predefined algorithms to adjust the harvesting header height, forming a closed-loop control system.

Figure 3c depicts the workflow diagram for a machine vision-based system. This system employs a camera to capture crop images, calculating plant height in real-time through offline-trained models. The controller ultimately adjusts the harvesting header to the optimal harvesting height.

2. Application status

(1) Ultrasonic sensor

The earliest sensors used for distance measurement were ultrasonic sensors, which operate by calculating the time difference between the emission and return of ultrasonic waves to determine the distance to the target object. Davinder [55] compared various models of ultrasonic sensors and developed an ultrasonic-based header detection system. However, as stubble density, sensor height, and harvester speed increase, the accuracy of all sensor types decreases. Sun [56] et al. designed a header height measurement and control system based on M18-type ultrasonic sensors. The system demonstrated good dynamic response characteristics and robustness, making it suitable for controlling header height in field environments. Cao [57] et al. proposed an automatic control system for the cutting header height of a corn harvester based on ultrasonic sensors. The system uses ultrasonic sensors to continuously monitor the cut-ting header height and can perform online self-tuning, demonstrating good robustness.

(2) LIDAR sensor

With the advancement of sensor technology, researchers have begun to explore the use of LiDAR sensors to measure header height in order to achieve more reliable control [58,59]. Zhang [60] et al. designed a face array LiDAR-based cutting header height adaptive system that can collect ground height data in real time within an 8 × 8 point array range, improving the accuracy of measurement results. The system has no overshoot phenomenon and can ensure efficient operation of the harvester in complex farmland terrain.

(3) Multi-sensor fusion

However, using a single sensor for measurement still has many drawbacks. Data from a single sensor may be subject to interference and produce outliers, making it unable to accurately reflect the actual height [61,62,63,64]. Therefore, a multi-sensor fusion-based method for measuring the height of the harvesting header has been introduced.

Long [65] et al. designed an adaptive height adjustment system for combine harvester cutting headers based on inclinometer sensors. By obtaining inclination data of the vehicle body and cutting header from multiple inclinometer sensors, a mathematical model is established to calculate the cutting header height in real time. Subsequently, an algorithm controls the extension or retraction of the electric push rod to achieve adaptive adjustment of the cutting header height. Shi [66] et al. designed an adaptive sugarcane cutting header height control system. Laser radar and wire displacement sensors are used to measure ground height and cut header height, respectively. The PLC control system then compares this height difference and actuates the hydraulic system to raise or lower the header accordingly. Yang [67,68] et al. addressed the issue that traditional leafy vegetable harvesters cannot adapt to changes in ridge surfaces by designing a multi-sensor fusion-based cutting header profiling control system. This system uses multi-point laser radar to obtain point cloud data of the leafy vegetable canopy, processes the point cloud data using machine learning models, and fits the optimal cutting plane to achieve automatic adjustment of the cutting header height. This cutting header profiling control system effectively enhances the intelligence level and operational quality of leafy vegetable harvesters, meeting the requirements of field harvesting operations for leafy vegetables.

(4) Machine vision

**Figure 4:**
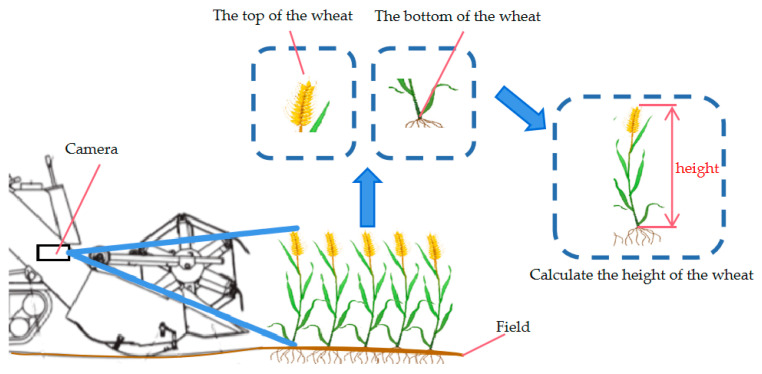
Schematic diagram of wheat plant height measurement based on images.

As illustrated in Figure 4, machine vision performs non-contact measurements using cameras, image-processing algorithms and computer hardware. This system captures multi-dimensional information such as color, texture and pattern to calculate plant height, and subsequently triggers the adjustment of the header height. [69].

Thanks to improvements in computer hardware performance, machine vision has also been widely applied and developed in the agricultural field [70]. Guo [71] designed a laser vision-based crop height measurement system. By combining an im-proved triangulation model and a specific checkerboard calibration method with laser vision technology, the system achieved automated and high-precision measurement of crop height, demonstrating high measurement accuracy and good practicality. Zeeshan [72] et al. designed a machine vision system based on deep learning for precise automated adjustment of the cutting height of wild blueberry harvesters. By using the YOLO deep learning model and a camera module, they developed a system capable of real-time detection of blueberry height and automatic adjustment of the cutting height. The system performs well in weed-free areas, accurately measuring blueberry height. However, in areas with dense weeds, the system’s performance is limited because weeds can obstruct blueberry fruits, increasing detection difficulty. Table 1 summarizes the performance of each sensor. The lidar sensor performs poorly compared to other sensors and is highly susceptible to environmental interference. Visual measurement results in smaller height errors and better performance.

#### 4.1.2. Contact Measurement

1. Structure and principle

**Figure 5:**
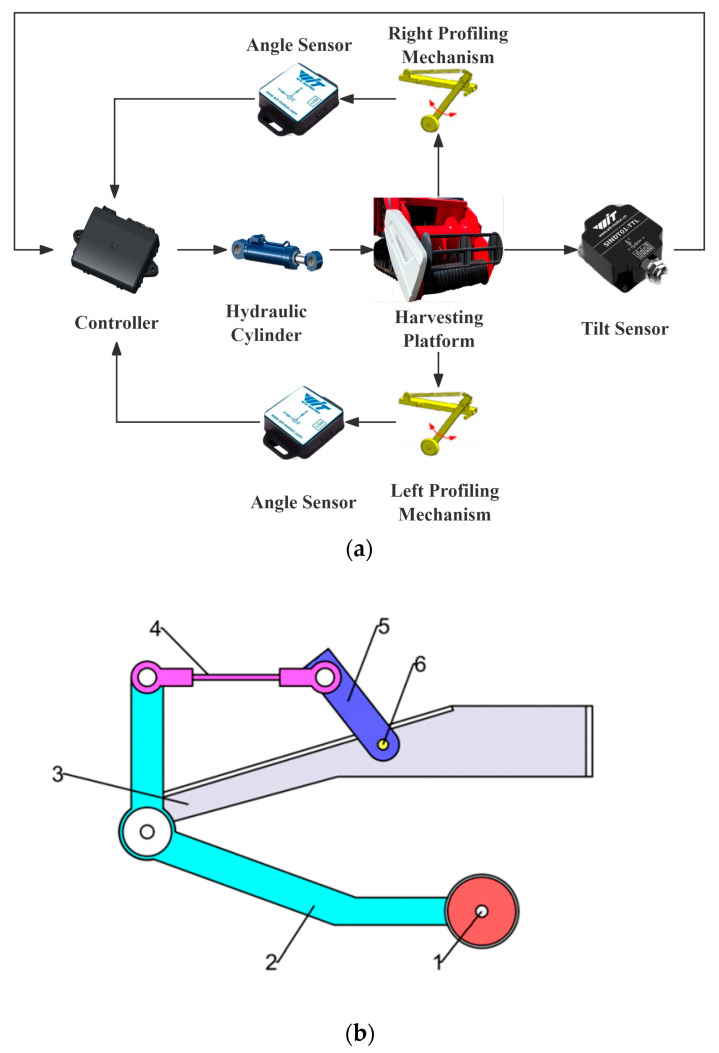
Schematic diagram of the contact height measurement workflow. (**a**) Contact height measurement workflow diagram; (**b**) Schematic diagram of the profiling device: 1. profiling wheel; 2. curved rod; 3. bracket; 4. adjustment screw; 5. sensor connecting rod; 6. angle sensor.

As shown in Figure 5a, contact harvesting header height measurement is achieved through the combination of a profiling device and sensors [73]. The profiling device consists of a profiling probe, a four-bar mechanism, and an angle sensor, as shown in Figure 5b. During operation, the profiling probe comes into contact with the farmland surface. Through the four-bar mechanism, the undulations of the farmland surface are converted into the rotational angle of the angle sensor [74,75]. The angle sensor transmits the signal to the controller, which precisely regulates the hydraulic cylinder based on the signal, enabling the harvesting header to operate at the optimal position. profiling devices can be classified into profiling rods (plates), single-wheel profiling devices, and dual-wheel profiling devices based on their structural design. Profiling plates cause minimal damage to crops, but the profiling rod may easily penetrate the ground during operation, and the profiling plate may cause soil displacement; dual profiling wheels provide stable operation but cause significant damage to crops; single profiling wheels offer stable operation with minimal damage to crops [76,77].

2. Application status

(1) Profiling rod (plate)

Wang [78] et al. designed an adaptive height control system for the harvesting header. By installing a profiling rod at the bottom of the harvesting header in conjunction with an angle sensor for height measurement and utilizing an electromagnetic proportional valve to achieve automatic adjustment of the harvesting header height, the system demonstrated excellent adaptive regulation capabilities. The stability and accuracy of the harvesting header height control significantly outperform traditional manual control methods. Wang [79] et al. designed an intelligent control system for grain harvesting header height based on multi-sensor data fusion, achieving stable control of the harvesting header height. The system demonstrated good control performance under different operating speeds and harvesting header heights, enhancing the intelligence level of the harvester. Wei [80] et al. designed a contact-type harvesting header height profiling control system. By calculating the actual height of the harvesting header based on the geometric relationship between the profiling rod and angle sensor, the system precisely controls the extension of the cylinder using an electro-magnetic directional control valve and displacement sensor, thereby achieving profiling control of the harvester on micro-topography.

Li [81] et al. designed a mechanical-hydraulic combination soybean harvesting header profiling device, which effectively improved the profiling range and flexibility of the soybean harvesting header through the combination of a profiling plate, cutter, and hydraulic cylinder. Zhang [82] et al. designed a height control system for corn harvesting header. The profiling plate works with an angle sensor to convert ground elevation changes into electrical signals, which are then used to control the cylinder to adjust the height of the harvesting header.

(2) Single and dual profiling wheels

Wang [83] et al. designed a soybean harvesting header profiling system. Angle sensors are used to measure changes in the angle between the profiling wheel and the ground, and the height of the cutter above the ground is calculated in real time. A hydraulic electromagnetic directional control valve is used to automatically control the lifting and lowering of the harvesting header. Tan [84] et al. designed a corn harvesting header height adaptive adjustment system, which uses pressure sensors installed on the profiling wheel to respond to ground undulations. Under the action of a spring, the pressure sensor has a pre-set pressure value. When the ground rises, the spring is compressed, increasing the pressure, and vice versa. Luo [85] designed a harvesting header profiling device with two degrees of freedom. This device can simultaneously adjust the height and tilt angle of the harvesting header, avoiding uneven stubble after cutting. Zheng [86] et al. designed an adaptive control system for the cutting header height of a rice harvester, using a wheel profiling device installed at the front of the cutting header. This control system has good stability and high reliability. Song Yang [87] et al. designed a ground height profiling detection device and harvesting header height control system. Angle sensors and inclinometers are used to calculate the height of the harvesting header above the ground. By combining small-amplitude fine adjustments with large-amplitude rapid adjustments, real-time control of the harvesting header’s height above the ground is effectively achieved. Cheng [88] uses profiling wheels and angle sensors to continuously monitor the relative posture between the harvesting header and the ground, ensuring it remains parallel to the ground and maintains the set stubble height. If the deviation on either side exceeds the threshold, the PLC outputs a signal to adjust the cylinder’s movement. As summarized in Table 2, the three profiling devices exhibit comparable average errors. However, profiling wheel-based systems consistently achieve the smallest errors and highest precision among them.

**Figure 6:**
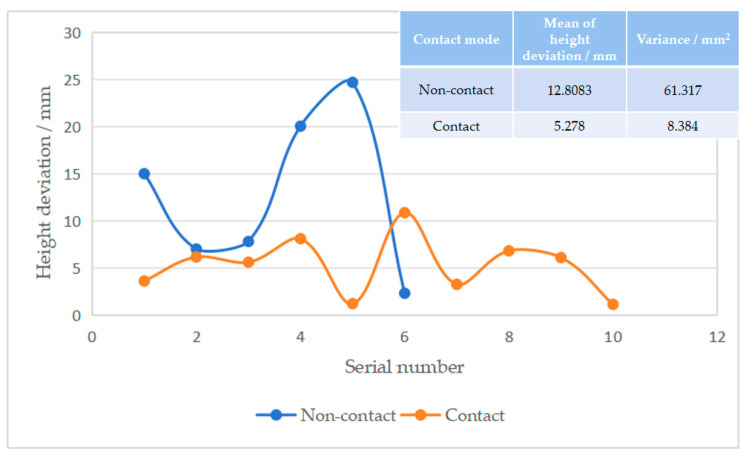
Comparison of contact and non-contact height measurement data.

Figure 6 compares the average errors of contact and non-contact height measurements. It can be seen that both the average error and variance of contact height measurements are smaller than those of non-contact measurements, indicating greater robustness in contact-based height measurement. From the perspective of error sources, contact height measurement directly engages with the ground surface, making it less susceptible to environmental factors and enabling more accurate representation of actual height. In contrast, non-contact height measurement exhibits high environmental dependency; factors such as strong light, shadows, dust, and debris can interfere with the sensor, leading to significant deviations between measured and actual values.

### 4.2. Control Algorithms of the Working Height of a Crop Harvesting Header

**Figure 7:**
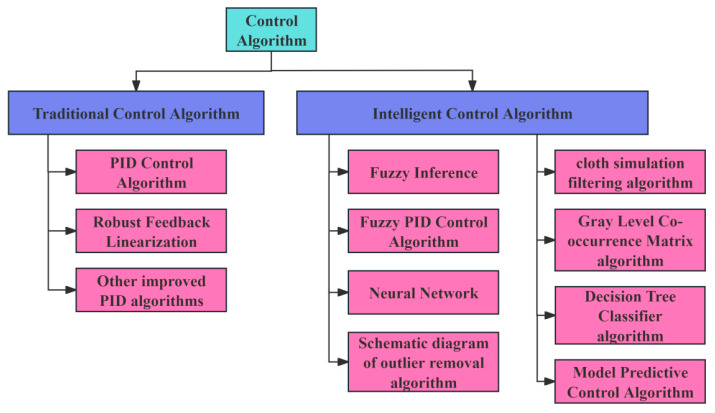
Classification diagram of control algorithms.

For an efficient, stable, and reliable harvesting header height control system, a smooth and responsive profiling device serves as the hardware foundation, while the quality of the control algorithm is equally critical. The control algorithm determines system performance and reduces system errors [89,90,91]. Currently, the algorithms used for harvesting header profiling control are primarily traditional PID control algorithms or improved versions based on them. Traditional control algorithms have low computational complexity and are highly mature in industrial applications, but they are generally only suitable for linear time-invariant systems and struggle to handle situations requiring constraint conditions [92]. As a result, researchers have begun to explore intelligent algorithms such as model predictive control, neural networks, and adaptive control. These control algorithms can handle multi-input/multi-output, nonlinear, time-varying, and strongly coupled systems. However, these intelligent algorithms remain experimental and are not yet mature for widespread application. The classification of harvesting header control algorithms is shown in Figure 7.

#### 4.2.1. Traditional Control Algorithms

1. Traditional PID control algorithms and their improved algorithms

**Figure 8:**
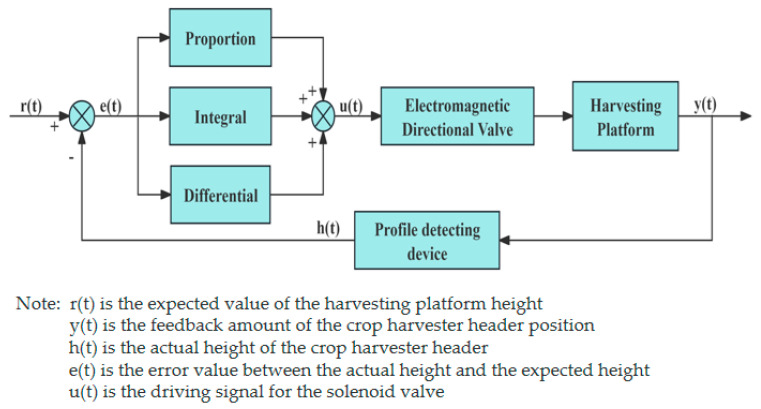
Schematic diagram of the PID algorithm.

The PID algorithm, as shown in Figure 8, is a widely used closed-loop control method in industrial applications. It stabilizes the system output by combining proportional, integral, and derivative actions to minimize deviation from the setpoint. Geng [93] et al. designed a PID-based adaptive system for adjusting the height of the harvesting header of a corn harvester to address issues such as inconvenient height adjustment. The STM32F4 is used as the main control unit to collect electrical signals from the angle sensor and control the electromagnetic directional valve to adjust the rise and fall of the harvesting header.

**Figure 9:**
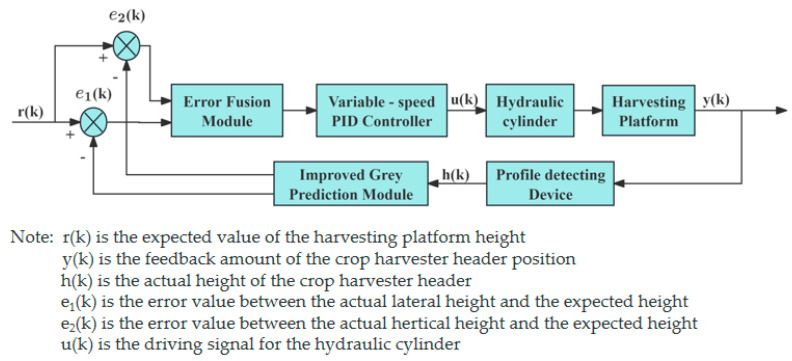
Schematic diagram of the improved PID algorithm.

The improved gray prediction PID algorithm was developed to overcome limitations of traditional PID control, such as slow response and poor adaptability to complex, dynamic harvester operating environments. While maintaining the original dimensionality of the model, the latest data is continuously integrated into the model, while the oldest data points are gradually phased out, thereby further improving control accuracy. The principle is illustrated in Figure 9. Huang [94] et al. designed a longitudinal height-lateral tilt cooperative harvesting header profiling height control system based on an improved gray prediction variable-speed PID. The improved system exhibits no significant overshoot, with stubble deviation reduced by 66.3% and pass rate increased by 42.42%, meeting operational requirements.

The EVPIVS-PID control algorithm can reduce the integral term parameter when the deviation is too large to avoid excessive overshoot of the system. Furthermore, different parameters are assigned to different speed ranges, thereby improving the response speed of the system. Yang [95] et al. designed a harvesting header height control system based on the EVPIVS-PID algorithm. This system can mitigate the integral accumulation speed under large deviation conditions, enabling the harvester to operate with lower fluctuations and higher stability at different operating speeds.

2. Robust feedback linearization control algorithm

**Figure 10:**
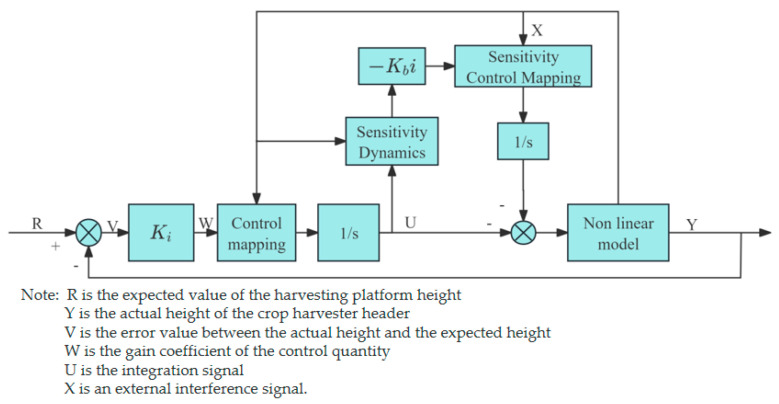
Schematic diagram of the robust feedback linearization.

Robust feedback linearization, illustrated in Figure 10, merges feedback linearization with robust control to enhance the performance and stability of nonlinear systems under uncertainty and disturbance. Zhuang [96] et al. established a mathematical model of the height control system based on the structure and dynamics analysis of the harvesting header, transforming the complex, multi-variable nonlinear system into a traditional linear system. They reduced the impact of uncertain parameters by constructing sensitivity dynamic equations and adjusting the control input.

#### 4.2.2. Intelligent Control Algorithm

Traditional control algorithms are already capable of satisfying the profiling control requirements of harvesting headers. To further reduce errors, researchers have proposed the use of intelligent control algorithms such as fuzzy PID control, combining expert control, fuzzy reasoning, and deep learning techniques.

1. Fuzzy inference control algorithm

Fuzzy reasoning is a reasoning technique based on fuzzy logic and fuzzy set theory that can handle uncertain, fuzzy, or semantically unclear information to make reasonable and reliable inferences and decisions. It is widely applied in numerous fields such as artificial intelligence. Cheng [97] et al. established a mathematical model through dynamic analysis of the vehicle body and harvesting header and designed a fuzzy control system for harvesting header height using the Mamdani fuzzy inference method. However, this system was only simulated and not tested in the field.

2. Fuzzy PID control algorithm

**Figure 11:**
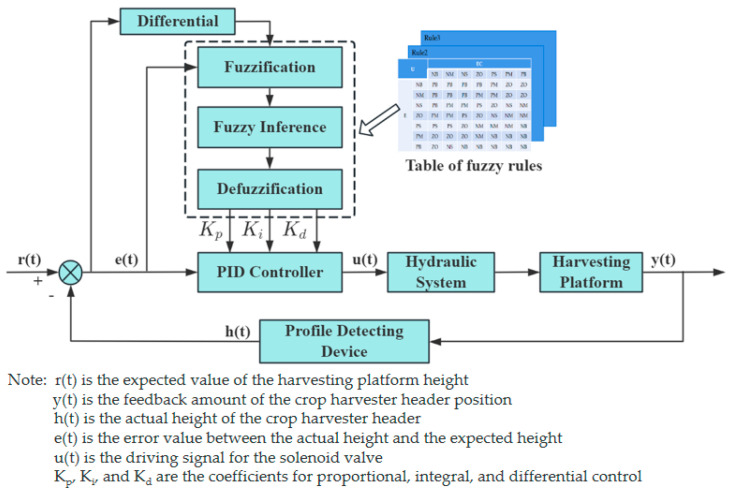
Schematic diagram of the fuzzy PID algorithm.

Fuzzy PID control is a control theory and technology that combines fuzzy logic with traditional PID control. The principle diagram is shown in Figure 11, and it is widely applied in fields such as industrial control. Its principle is to adjust the coefficients of the PID controller in real time based on system error and error rate of change using fuzzy logic to adapt to system changes and improve control performance. Liu [98] et al. designed a fuzzy PID-based adaptive height profiling control system for rice harvesters. The system was validated through bench tests and simulations. Yao [99] et al. designed an automatic row and height control system for a harvester based on a monocular camera. By extracting feature points from the edges of wheat, the current height of the harvesting header is calculated and compared with the set parameters. Ultimately, the fuzzy PID algorithm controls the hydraulic actuator to achieve the lifting and lowering of the harvesting header.

**Figure 12:**
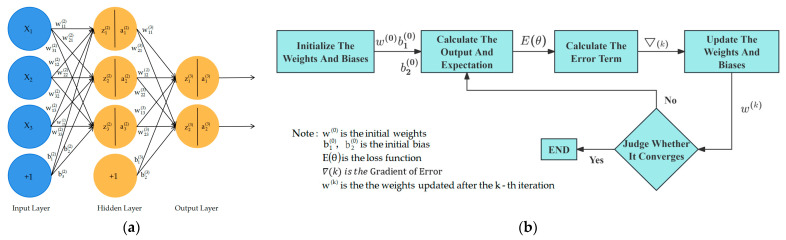
Schematic diagram of the neural network. (**a**) Schematic diagram of a three-layer neural network structure; (**b**) Backpropagation principle diagram of neural networks.

Neural networks are an important algorithm in the field of intelligent computing. They primarily optimize parameters through the error backpropagation algorithm, making the parameter calculations for neurons in multi-layer neural networks simpler. Figure 12 presents a schematic of a neural network, a is the structure of a three-layer network, and b is the backpropagation process. Zhang [100] et al. designed a harvesting header profiling control system based on the fusion processing of ultrasonic arrays and angle sensors. By using neural networks and digital filters to filter and fuse sensor measurement values, they improved measurement accuracy, enhanced the correlation between sensors, and avoided the limitations of single-sensor measurements. Finally, they precisely controlled the electromagnetic directional valve using a fuzzy PID algorithm.

3. IDBO-PID control algorithm

**Figure 13:**
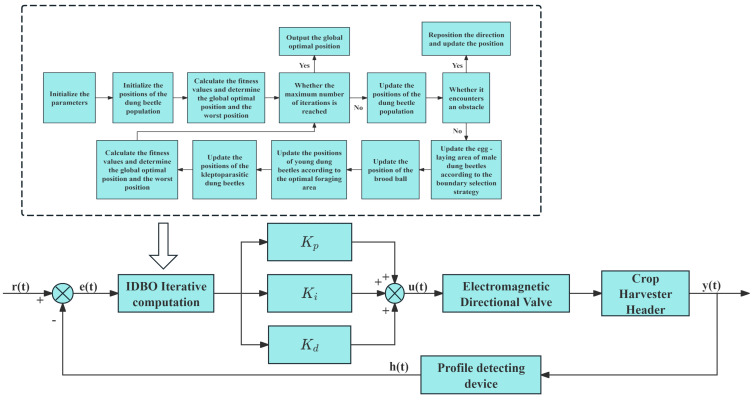
Schematic diagram of the IDBO-PID algorithm.

The DBO-PID algorithm iteratively calculates optimal control parameters by simulating the behaviors of dung beetles, such as rolling balls, dancing, foraging, and reproducing. However, it has slow convergence speed and is prone to getting stuck in local optima. The IDBO-PID algorithm addresses these issues by introducing a chaotic mapping strategy to replace the DBO random operator, thereby enhancing search capabilities. The principle diagram is shown in Figure 13. Zhang [101] et al. designed a harvesting header height control system using an optimized IDBO-PID algorithm. The system exhibits nearly zero overshoot, with response speed and convergence speed significantly faster than those of traditional PID algorithms.

**Figure 14:**
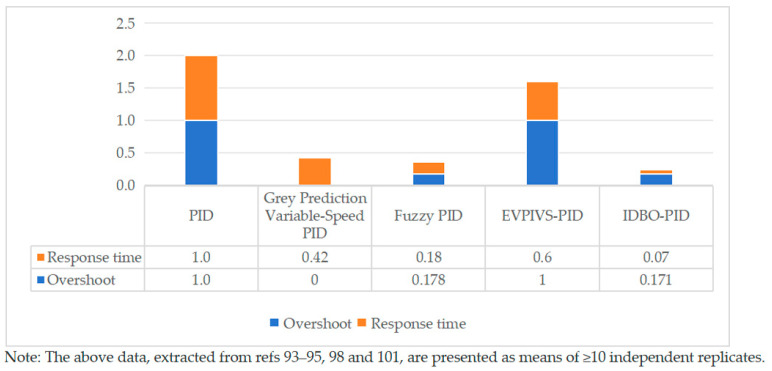
Comparison of data from different PID algorithms.

Figure 14 compares four improved PID algorithms with the PID algorithm as a reference (Data 0 indicates that under the same control system, using this algorithm does not cause overshoot, while using the PID algorithm does cause overshoot. Data 1 indicates that under the same control system, neither this algorithm nor the PID algorithm causes overshoot). The fuzzy PID algorithm and IDBO-PID algorithm achieve response speeds 58% and 93% faster than the traditional PID algorithm, outperforming the other two algorithms. In terms of overshoot, the Grey prediction speed change PID algorithm and EVPIVS-PID algorithm ensure the system has no overshoot, while the other two intelligent algorithms result in minor overshoot. Overall, intelligent algorithms are more sensitive and better suited for harvester operations, as they can respond more quickly to changes in the ground surface and improve profiling accuracy.

4. Outlier removal algorithm and cloth simulation filter algorithm

**Figure 15:**
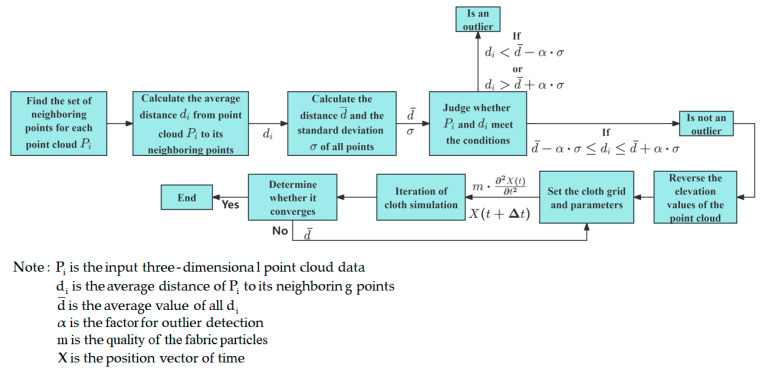
Schematic diagram of the outlier removal algorithm and cloth simulation filter algorithm.

Outlier removal algorithms are statistical methods that can identify and remove abnormal data. Cloth simulation filter algorithm is filtering methods based on simulated fabrics, mainly used for point cloud data processing, as shown in Figure 15. Xie [102] et al. designed an adaptive harvesting system based on visual segmentation of the lodged area and simultaneous extraction of multiple feature information (lodged position, direction, etc.). Infrared images are used to detect and obtain the areas and boundaries of fallen crops. The outlier removal algorithm and cloth simulation filter algorithm are then applied to calculate the relative height difference between the fallen and non-fallen areas, thereby establishing a segmentation reference plane between the two. This system exhibits excellent stability and robustness, ensuring high efficiency and low loss during harvester operations.

5. Grayscale coexistence matrix algorithm and decision tree classifier algorithm

The grayscale co-occurrence matrix algorithm is a commonly used method for describing image texture features, reflecting image texture information by studying spatial correlation characteristics. The decision tree classifier is a simple and easy-to-use nonparametric classifier, often used to solve classification problems. Liu [103] et al. designed a vision-based harvesting header height prediction system. The system analyzes the field area in front of the harvester to predict when the harvesting header needs to be raised to avoid wasting crops or damaging equipment. The system first segments the field area using motion consistency and optical flow, followed by fine segmentation via color distribution. Texture features are then extracted using a grayscale co-occurrence matrix and classified with a decision tree to distinguish crops from empty areas. Finally, it generates a probability map, computes the crop percentage per frame, and fits a trend function to predict the optimal time to raise the header. The system demonstrated robust performance across varying lighting and field conditions, achieving high accuracy (0.902 for soybeans, 0.935 for wheat). However, it is limited to predicting only the raise time, not the lower time, and segmentation inconsistencies could propagate errors into the crop percentage calculations.

6. Model predictive control algorithm

**Figure 16:**
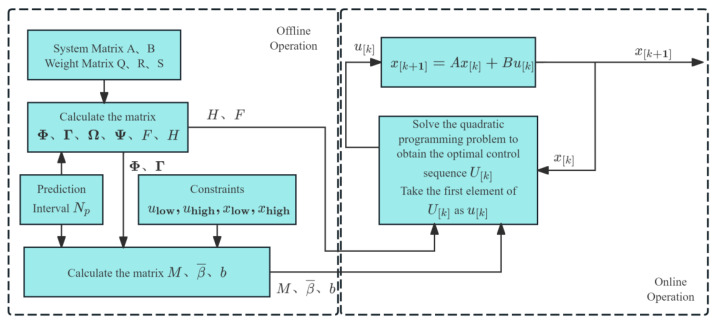
Schematic diagram of the model predictive control algorithm.

Model predictive control is an advanced control algorithm based on dynamic models, achieving optimal control of multi-variable systems through rolling optimization and feedback correction. The principle diagram is shown in Figure 16. Wang [104] proposed a model predictive control-based harvesting header height control algorithm, establishing a linear error model for the harvesting header height, designing and optimizing the objective function to achieve precise control of the harvesting header height. Under different operating speeds and harvesting header height conditions, the average height control accuracy was 91.5% and 91.25%, respectively. In all experiments, the harvesting header did not touch the ground, effectively addressing the interference caused by complex terrain on harvesting header height control.

As summarized in Table 3, intelligent control algorithms generally achieve faster response speeds than traditional ones, though they do not consistently yield smaller height errors.

From the perspective of resource requirements and applicable scenarios, traditional algorithms have low computational complexity and consume minimal data resources, making them suitable for development in resource-constrained embedded systems. However, traditional algorithms are unable to handle complex systems and struggle with multi-objective, multi-constraint problems. Intelligent control algorithms are highly adaptive, capable of dynamic adjustment, and have strong interference resistance. Some algorithms can even learn autonomously, provide early warnings, and adjust the system accordingly. However, intelligent algorithms require more computational resources, more data storage space, and higher hardware requirements, making them difficult to deploy in resource-constrained embedded systems.

## 5. Conclusions and Prospects

### 5.1. Conclusions

This review has systematically chronicled the evolution of intelligent control technology for harvesting header height, tracing its progression from early mechanical systems to modern sensor- and controller-centric electro-hydraulic profiling. In sensing, both non-contact and contact methods offer distinct advantages: non-contact approaches, particularly machine vision, provide high precision without ground interference, while contact methods deliver superior reliability and lower error. At the control level, traditional PID and its variants remain prevalent due to their simplicity and maturity. However, intelligent algorithms are demonstrating significant potential for managing the complex, nonlinear dynamics of farmland, presenting a viable path toward high-speed, high-precision operation. Overall, advancements in multi-sensor fusion and intelligent control are substantially boosting agricultural productivity and sustainability, accelerating the transition toward intelligent, precision agricultural systems.

### 5.2. Prospects

Although significant progress has been made in the intelligent control technology for harvesting header operating height, electro-hydraulic profiling control still has certain limitations in some aspects. These mainly include the following:

1. Insufficient measurement accuracy of working height in complex terrain. Soil conditions and planting patterns vary significantly across different regions. Traditional profiling devices are typically rigid structures, which struggle to fully conform to the ground when operating in terrain with significant undulations, steep slopes, or soft ground. Under such extreme conditions, sensors may exhibit sluggish responses and significantly increased errors. It is necessary to optimize the manufacturing materials of profiling devices, replacing existing materials with shape memory alloys such as nickel-titanium alloys. By leveraging their self-responsive properties (e.g., force feedback), these materials can rapidly and reversibly transition between “soft” and “rigid” states, When encountering a trench, the profiling device is subjected to upward force, inducing stress-induced martensite transformation that softens the entire structure, causing the device to actively sink and adhere to the ground. Upon encountering an elevated object, external force is released, instantly restoring the austenitic state. The profiling device then rebounds, maintaining ground contact pressure.
2. Slow response and large delay of working height hydraulic control system. Hydraulic systems exhibit slow response times, low energy efficiency, and complex system structures. Typical hydraulic systems achieve transmission efficiencies of around 70%, with response times exceeding 50 ms. Additionally, hydraulic systems require numerous auxiliary components such as reservoirs, filters, coolers, accumulators, and intricate piping networks. Maintenance complexity is high, necessitating regular replacement of hydraulic fluid and filter elements, along with leak inspections. In contrast, electric drive systems achieve over 90% efficiency, a 28% improvement. with response times under 10 ms, an 80% reduction. They require far fewer components than hydraulic systems, ensuring significantly lower failure rates.
3. Incompatibility between working height control models and strategies. Simple kinematic models are only applicable under low-speed conditions, while designing dynamic models results in increased computational complexity. Developing an accurate and easily controllable control model is the future direction of research. Current research primarily focuses on low-speed operation, and existing control strategies poor robustness at high speeds. In practical applications, a single control strategy may have certain drawbacks. In the future, algorithms such as PID control and model predictive control can be integrated. By tuning the weighting coefficients of the MPC control algorithm through PID tuning, the system can both account for past errors and predict future height. Leveraging their complementary advantages will enhance system stability.
4. The height measurement method is limited to a single approach. Non-contact height measurement is susceptible to environmental factors such as lighting and dust; contact-based height measurement is affected by protrusions like hard soil clumps, leading to increased errors. In the future, integrating both measurement methods through techniques like complementary filtering and adjusting the confidence level for each scenario could effectively mitigate their respective drawbacks and reduce measurement errors.

## Figures and Tables

**Table 1:** Non-contact height measurement.

Crop	Height Measurement Sensor	Performance	References
Buckwheat	Ultrasonic sensor	The average error in the height of the harvesting header above the ground is 15 mm	[56]
Corn		The average error in the height of the harvesting header above the ground is 7 mm	[57]
Rice	Tilt sensor	The average error in the height of the harvesting header above the ground is 7.8 mm	[65]
Sugarcane	LIDAR sensor Displacement sensor	The average error in the height of the harvesting header above the ground is 20 mm	[66]
Leafy vegetables	LIDAR sensor	The average error in the height of the harvesting header above the ground is 24.7 mm	[67,68]
Blueberries	Camera module	The average error in the height of the harvesting header above the ground is 2.3 mm	[72]

**Table 2:** Contact height measurement.

Crop	Type of Profiling Device	Performance	References
Rice	Profiling rod	The average error in the height of the harvesting header above the ground is 3.6 mm	[79]
Rice		The average error in the height of the harvesting header above the ground is 6.15 mm	[80]
Soybeans	Profiling plate	The average error in the height of the harvesting header above the ground is 5.6 mm	[81]
Corn		The average error in the height of the harvesting header above the ground is 8.1 mm	[82]
Soybeans	profiling wheel	The average error in the height of the harvesting header above the ground is 1.2 mm	[83]
Corn		The average error in the height of the harvesting header above the ground is 10.86 mm	[84]
Grass		The average error in the height of the harvesting header above the ground is 3.25 mm	[85]
Rice		The average error in the height of the harvesting header above the ground is 6.81 mm	[86]
Rice		The average error in the height of the harvesting header above the ground is 6.09 mm	[87]
Grass		The average error in the height of the harvesting header above the ground is 1.12 mm	[88]

**Table 3:** Performance data and applicable scenarios for the harvesting header height control algorithm.

Arithmetic	Response Time/s	Average Error /mm	Applicable Scenarios	References
PID	/	8.25	Simple linear control system	[93]
Grey prediction speed change PID	2	8.68	Slow time-varying, small lag system	[94]
EVPIVS-PID	/	2.95	Systems subject to significant external interference and integration saturation	[95]
Robust feedback linearization	/	4.65	Nonlinear systems with model uncertainty and external disturbances	[96]
Fuzzy PID	1.68	6.75	Nonlinear systems with certain requirements for response speed	[98]
IDBO-PID	1.11	5.4	Complex multivariable, strongly coupled systems	[101]
Outlier removal	/	2.5	A system with high data quality requirements, large data scale, and complex abnormal situations and dynamic simulation of fabric effects in virtual scenes	[102]
Cloth simulation filter				
Model predictive control	0.625	4.25	A system with multiple variables, nonlinearity, and high requirements for control accuracy and real-time performance	[104]

## Data Availability

The data presented in this study can be obtained from the first author.
